# Leprosy in Sonora, Mexico: A 14-Year Retrospective Study of Clinical, Epidemiological, and Sociodemographic Characteristics

**DOI:** 10.3390/tropicalmed11100278

**Published:** 2026-09-30

**Authors:** Eliezer Emmanuel Preciado Gocobachi, Miguel Alberto Salgado Salomón, Gonzalo Armando Navarro Armendariz, Sergio Trujillo López, Sayil Alejandra De la Torre Othón, Jurgen Adam Sánchez, Gerardo Álvarez Hernández, Jorge Laureano Eugenio

**Affiliations:** 1 Departamento de Medicina y Ciencias de la Salud, Universidad de Sonora, Hermosillo 83000, Mexico; 2 Secretaría de Salud Pública de Sonora, Hermosillo 83000, Mexico

**Keywords:** leprosy, Hansen’s disease, Sonora, *Mycobacterium leprae*, retrospective study

## Abstract

Leprosy remains a public health challenge due to delayed diagnosis, disability, and persistent transmission despite the availability of effective treatment. This study aimed to describe the sociodemographic, clinical, and epidemiological profile of leprosy in the state of Sonora, Mexico, from 2011 to 2024. A descriptive, observational, retrospective study was conducted using data from the state leprosy registry. Sociodemographic, clinical, and epidemiological variables were analyzed through descriptive statistics, and annual incidence and prevalence rates were calculated. A total of 78 cases were identified during the study period. Patients had a mean age of 43.9 years, and sex distribution was nearly equal. Most cases occurred in urban areas, with Hermosillo and Cajeme accounting for the largest proportions. Lepromatous leprosy was the predominant clinical form (61.3%), and 72% of patients were classified as multibacillary. Disability at diagnosis was present in 66.6% of patients, while the mean interval from symptom onset to diagnosis was 47.8 months. Annual incidence and prevalence rates remained low but fluctuated throughout the study period, with new cases reported every year. Most patients reported probable acquisition within Sonora, and routine medical consultation was the main mode of case detection. These findings indicate that delayed diagnosis and disability remain major challenges in Sonora, highlighting the need for improved early detection strategies, strengthened surveillance, and enhanced medical training in leprosy.

## 1. Introduction

Leprosy, also known as Hansen’s disease, is a chronic infectious disease caused by the *Mycobacterium leprae* complex, which comprises *M. leprae* and *M. lepromatosis*. It primarily affects the skin and peripheral nerves [1,2].

Despite the availability of diagnostic tools and effective treatment, transmission persists in several countries and continues to represent a public health challenge due to delayed diagnosis, disease-associated disability, and social stigma [3,4]. According to the World Health Organization (WHO), an average of 200,000 cases were reported in 2019, with a predominance of multibacillary forms and a relevant proportion of patients presenting with disability at the time of diagnosis, suggesting delays in case detection and ongoing active transmission [5,6].

In Mexico, both the incidence and prevalence of leprosy are concentrated mainly in the western and northeastern regions of the country [7]. In the state of Sonora, leprosy occurs in a low-endemic setting. According to WHO technical guidance, low-endemic areas are those reporting fewer than 50 new cases per million population per year [8]. The disease remains a societal challenge due to its social, economic, and health-related impact on affected individuals. Although studies conducted in Mexico have described the profiles of patients in both urban and rural settings, there is a lack of systematically compiled information regarding cases registered in the state of Sonora [9,10]. The absence of consolidated data highlights the need for a retrospective analysis to characterize the disease within the Sonoran population, identify its patterns, and provide evidence to support improvements in prevention, diagnosis, and treatment.

The aim of this study was to describe the sociodemographic, clinical, and epidemiological profile of leprosy in the state of Sonora during the period 2011–2024.

## 2. Materials and Methods

A descriptive, observational, and retrospective study was conducted in the state of Sonora, Mexico. The study population consisted of leprosy cases recorded in the state database of the Secretaría de Salud between January 2011 and December 2024. Case information was reported through the state epidemiological surveillance system by healthcare facilities, including primary healthcare centers and hospitals at different levels of care, which included Secretaría de Salud, Instituto Mexicano del Seguro Social (IMSS), Instituto de Seguridad y Servicios Sociales de los Trabajadores del Estado (ISSSTE), Instituto de Seguridad y Servicios Sociales de los Trabajadores del Estado de Sonora (ISSSTESON), as well as other facilities in the state. Cases were defined according to the Mexican operational criteria. Cases were defined according to the operational criteria established in the Mexican Official Standard NOM-027-SSA2-2007 [11]. A probable case was defined as any person presenting hypopigmented, reddish, or copper-colored macules with sensory impairment, or infiltrated plaques, nodules, diffuse infiltration, ulcers, or areas of sensory impairment without dermatological lesions. A confirmed case was defined as a probable case with a positive slit-skin smear; when bacilloscopy was negative, confirmation required epidemiological evidence and histopathological findings compatible with leprosy. According to the national diagnostic protocol, bacilloscopy is performed on slit-skin smears obtained from the earlobe and a cutaneous lesion and, when indicated, from the nasal mucosa. Acid-fast bacilli are assessed using Ziehl–Neelsen staining [11]. Histopathological evaluation is performed on a skin biopsy obtained from an active lesion and including subcutaneous tissue. Routine tissue morphology is evaluated using hematoxylin and eosin staining, whereas Fite–Faraco staining is used to demonstrate *M. leprae* and *M. lepromatosis* in tissue sections [12]. Because this study was based on secondary registry data, the specific histochemical stains used for each individual specimen were not available in the analytical database.

The inclusion criteria were: (1) a confirmed diagnosis of leprosy according to the Mexican operational definition; (2) registration in the Sonora state leprosy surveillance database between 1 January 2011 and 31 December 2024; and (3) availability of sufficient information for the corresponding analysis. Records were not excluded from the study solely because of partially missing information. Instead, cases with incomplete or inconsistent data were excluded only from analyses requiring the missing variables. Cases with sufficient information on year of diagnosis and geographic location were retained for epidemiological analyses of incidence, prevalence, and temporal distribution. Records were excluded entirely from a specific analysis only when the information required for that analysis was unavailable or inconsistent. For the analysis of case-detection methods, “contact examination” was defined as the clinical evaluation of household or other close contacts of a previously diagnosed patient with leprosy to identify signs or symptoms suggestive of the disease.

A descriptive analysis of the sociodemographic, clinical, and epidemiological variables was performed. Absolute frequencies and percentages were calculated for categorical variables, whereas measures of central tendency and dispersion were determined for continuous variables according to the distribution of the data. Annual incidence and prevalence rates per 1,000,000 inhabitants were calculated based on population projections provided by Consejo Nacional de Población and Instituto Nacional de Estadistica y Geografía (CONAPO/INEGI). For spatial analysis, cumulative municipal incidence was calculated by dividing the total number of leprosy cases reported in each municipality during the period by the corresponding population, and was expressed as cases per 1,000,000 inhabitants. Data analysis was performed using Microsoft Excel and QGIS 4.2.2 software.

This study employed an observational, retrospective design based on the analysis of previously recorded secondary data sources. It involved no intervention on patients and no direct contact with them at any stage of the research process; therefore, it was classified as a minimal-risk study in accordance with the Mexican General Health Law on Health Research.

## 3. Results

The database used for the present study included all patients diagnosed with leprosy in the state of Sonora between 1 January 2011 and 31 December 2024. During this period, a total of 78 new cases were identified. Three records were found to contain incomplete information, particularly regarding sociodemographic and clinical variables. Consequently, one case from 2011 and two cases from 2012 were excluded from these specific analyses.

However, these exclusions were partial and did not affect the epidemiological analysis of the study (incidence and prevalence rates). Therefore, all 78 registered cases were included in the epidemiological assessment, as the three aforementioned records contained sufficient information for the evaluation of temporal distribution and disease trends. This approach preserved the integrity of the population-based analysis and provided a more comprehensive characterization of leprosy occurrence in the state of Sonora.

### 3.1. Sociodemographic Profile

Sex distribution was nearly equal, with 50.66% of patients being male and 49.33% female. Age ranged from 13 to 84 years, with a mean age of 43.9 ± 15.8 years. The age distribution showed a predominance of patients between 30 and 59 years of age, particularly in the 40–49-year age group, which accounted for 25.3% of all cases. Most patients were employed (82.66%), whereas 17.33% were unemployed. Urban residence predominated, accounting for 84% of cases, while 16% resided in rural areas (Table 1).

The geographic distribution of leprosy cases showed that Hermosillo accounted for the highest proportion of cases (25.3%, *n* = 19), followed by Cajeme (24.0%, *n* = 18) and Navojoa (10.7%, *n* = 8). The remaining cases were distributed among several municipalities, each contributing a smaller proportion of the total.

When cumulative incidence rates were adjusted for the population of each municipality, a different geographic pattern emerged. La Colorada had the highest cumulative incidence (541.13 cases per 1,000,000 inhabitants), followed by General Plutarco Elías Calles (366.92 per 1,000,000) and Baviácora (313.38 per 1,000,000). Elevated incidence rates were also observed in Ímuris, San Ignacio Río Muerto, Navojoa, Benito Juárez, Bácum, and Cajeme (Figure 1).

### 3.2. Clinical Profile

The most common clinical form was lepromatous leprosy, observed in 61.3% of patients, followed by indeterminate leprosy in 17.3%. Tuberculoid and borderline forms each accounted for 10.7% of cases. Overall, 72% of patients were classified as multibacillary leprosy, whereas 28% were classified as paucibacillary leprosy.

Regarding cutaneous manifestations, hypopigmented macules were present in 50.7% of patients, with more than five lesions being the most common presentation (45.3%). Erythematous–coppery macules were observed in 38.7% of cases, while nodules were present in 40%, frequently involving more than five lesions. Infiltrated plaques were identified in 22.7% of patients. Diffuse infiltration was uncommon and was observed in only 2.7% of cases (Table 2).

Regarding neurological manifestations, neuritis was documented in 4% of patients. However, anesthetic areas were a frequent finding, occurring in 60% of cases, and nearly half of the patients (46.7%) presented with more than five affected areas (Table 2).

At the time of diagnosis, 33.3% of patients had no disability (grade 0), whereas 50.6% had grade 1 disability and 16% had grade 2 disability. Overall, 66.6% of patients presented with some degree of disability at diagnosis.

During the course of the disease, 16% of patients experienced at least one episode of leprosy reaction. All patients with leprosy reactions developed type 2 reactions, while two patients (16.7%) also experienced type 1 reactions (Table 2).

Regarding diagnostic investigations, slit-skin smear microscopy was performed in all patients and yielded positive results in 50.7% of cases. Histopathological examination was also performed in all patients and demonstrated findings consistent with lepromatous leprosy in 61.3%, indeterminate leprosy in 17.3%, borderline leprosy in 10.7%, and tuberculoid leprosy in 10.7%.

The median interval between symptom onset and diagnosis was 35 months (IQR: 13–55.5), with a mean of 47.8 ± 57.6 months, ranging from 1 to 313 months. Only 24% of patients were diagnosed within the first 12 months after symptom onset, whereas 76% experienced a diagnostic delay exceeding 12 months (Table 3).

Regarding patient outcomes, 43 of the 75 patients (57.3%) were recorded as cured, while 13 (17.3%) remained under treatment or follow-up at the time of the last available record. Seven patients (9.3%) had died, five (6.7%) were lost to follow-up, one (1.3%) had abandoned treatment, and the outcome was unknown for six patients (8.0%). Treatment and follow-up were provided primarily at the primary healthcare level (72.0%), followed by secondary (24.0%) and tertiary care (4.0%) (Table 4).

### 3.3. Epidemiological Profile

Annual incidence showed a fluctuating pattern throughout the study period. The highest incidence rate was recorded in 2014, reaching 4.88 cases per 1,000,000 inhabitants, whereas the lowest rate was observed in 2023, at 0.32 cases per 1,000,000 inhabitants (Figure 2).

Annual prevalence also varied over time. The highest prevalence was observed in 2012, at 9.32 cases per 1,000,000 inhabitants. In subsequent years, prevalence fluctuated, reaching 5.17 cases per 1,000,000 inhabitants in 2015 and increasing to 7.53 in 2016, the second-highest value observed during the study period. From 2017 onward, prevalence generally remained below the 2016 level, with values of 6.12 in 2017, 5.07 in 2018, 3.35 in 2019, and subsequent fluctuations, reaching 6.43 cases per 1,000,000 inhabitants in 2024 (Figure 3).

Regarding the probable place of infection acquisition, 78.7% of patients reported probable acquisition within the state of Sonora, whereas 21.3% were considered to have acquired the infection outside the state. The most common method of case detection was routine medical consultation, through which 86.7% of cases were diagnosed, while 13.3% were identified through contact examination. Diagnosis was established primarily at the primary healthcare level (70.7%), followed by secondary (22.7%) and tertiary care facilities (6.7%).

## 4. Discussion

The sociodemographic profile showed a nearly homogeneous distribution between sexes, differing from national reports in which leprosy more frequently affects males [13,14]. Male sex has also been associated with multibacillary disease and higher grades of disability [15]. Most patients were between 30 and 59 years of age, highlighting the potential functional and socioeconomic impact of the disease among the economically active population [16,17].

Most patients resided in urban areas (84%), whereas 16% resided in rural areas. Hermosillo, Cajeme, and Navojoa accounted for the largest proportions of registered cases. However, population-based incidence or prevalence rates stratified by urban and rural residence were unavailable; therefore, these proportions do not allow conclusions regarding differences in disease occurrence or potential underdiagnosis between these settings. Differences in healthcare access may influence case detection, but this could not be evaluated with the available data.

From a clinical perspective, lepromatous and multibacillary forms predominated, with more than 60% of cases classified accordingly, consistent with previous reports [12,14]. Multibacillary disease is characterized by a higher bacillary burden and is associated with neurological involvement and leprosy reactions [12]. Extensive cutaneous lesions, including macules, nodules, and infiltrated plaques, have also been described more frequently in patients with multibacillary disease and higher bacillary loads [18]. Neuritis and anesthetic areas reflect the characteristic neural involvement of leprosy and are clinically relevant because delayed recognition may result in irreversible disability [19,20]. Diagnostic delay, however, may occur in both multibacillary and paucibacillary forms and should be considered independently of operational classification.

A major finding was the substantial diagnostic delay. Seventy-six percent of patients experienced a delay exceeding 12 months, with a mean of 47.8 months and a median of 35 months. The median observed in this study was near the upper end of the range reported internationally; Dharmawan et al. reported median delays ranging from 12 to 36 months and mean delays from 11.5 to 61.4 months [4]. In contrast, a study conducted in endemic areas of Brazil reported a median diagnostic delay of 15 months [21]. Diagnostic delay has been associated with an increased risk of disability [22], and in our population 66.6% of patients already had grade 1 or grade 2 disability at diagnosis. Thus, although leprosy has a low incidence rate in Sonora, diagnosis frequently occurs at an advanced stage. Low clinical suspicion, limited medical training, barriers to specialized healthcare access, and stigma have been identified as factors contributing to delayed diagnosis [4,21].

The fact that 70.7% of cases were diagnosed at the primary healthcare level highlights the importance of clinical recognition in this setting. In addition, only 13.3% of cases were detected through contact examination, whereas most were identified through routine medical consultation. This predominance of passive detection suggests an opportunity to strengthen clinical training, contact tracing, and active case-finding strategies, particularly among household and other close contacts. Previous evidence has also identified multibacillary and lepromatous disease as risk factors for physical disability [23], while a Mexican tertiary referral center reported lepromatous leprosy in approximately three-quarters of patients [14]. High frequencies of grade 2 disability have been considered by the WHO as an indicator of deficiencies in early detection and healthcare services [5,24].

From an epidemiological perspective, annual incidence remained low but fluctuated throughout the study period. The continued identification of new cases should not be interpreted as direct evidence of ongoing local transmission, given the prolonged incubation period and the fact that 21.3% of patients reported probable acquisition outside Sonora. The decline observed during the COVID-19 pandemic should also be interpreted cautiously, as disruptions in healthcare services affected leprosy detection and reporting [10,25].

The highest prevalence was observed in 2012, at 9.32 cases per 1,000,000 inhabitants. Unlike incidence, prevalence includes both newly diagnosed patients and previously diagnosed patients who remain registered as active cases during treatment or follow-up. Therefore, the 2012 peak may reflect an accumulation of active cases rather than an abrupt increase in transmission. The retrospective data available did not allow the specific contributions of treatment duration, follow-up, or case closure to this peak to be quantified. In addition, more than 20% of patients reported probable acquisition outside the state, and migration has been recognized as a factor that may influence the geographic distribution and control of leprosy [26,27].

This study has inherent limitations related to its retrospective design and dependence on the completeness and consistency of surveillance records. Detailed socioeconomic information, including household income, educational level, housing characteristics, and other socioeconomic indicators, was unavailable; therefore, socioeconomic status could not be assessed. Information on previous residence, migration history, and duration of residence was also not systematically recorded. In addition, patient-level information regarding the specific multidrug therapy regimen received and whether treatment was administered at home was not available in the analytical database; therefore, these aspects could not be evaluated retrospectively. Some variables may also have been subject to diagnostic heterogeneity across years and healthcare facilities. Nevertheless, this study provides one of the first comprehensive clinical and epidemiological characterizations of leprosy in Sonora over a 14-year period.

## 5. Conclusions

The diagnostic delay and disability rates observed in this cohort underscore the need for physicians in Sonora to continue considering leprosy in the differential diagnosis of dermatoses and neuropathies.

Updating and innovating detection strategies undoubtedly represents an important area of opportunity; however, medical training in leprosy should also constitute a fundamental pillar of education for healthcare professionals.

Although the present study was not designed to evaluate statistical associations between variables, these findings suggest the need for the development of new lines of research aimed at identifying factors associated with diagnostic delay and assessing their potential impact.

## Figures and Tables

**Figure 1:**
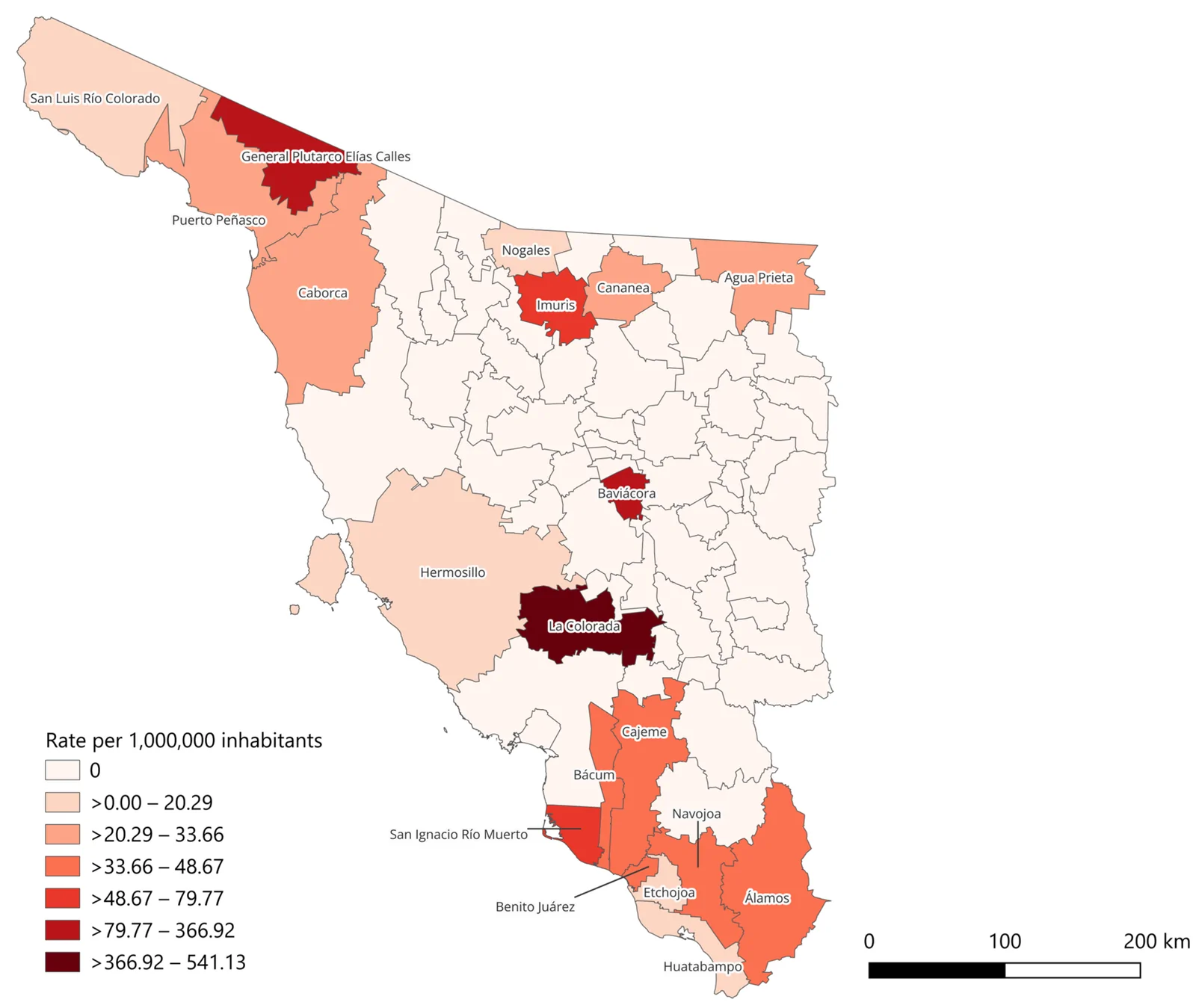
Geographic distribution of cumulative leprosy incidence by municipality in Sonora, Mexico, 2011–2024 (*n* = 75). Rates are expressed as cases per 1,000,000 inhabitants; darker shading represents higher cumulative incidence. Municipalities with the highest rates are individually labeled for clarity.

**Figure 2:**
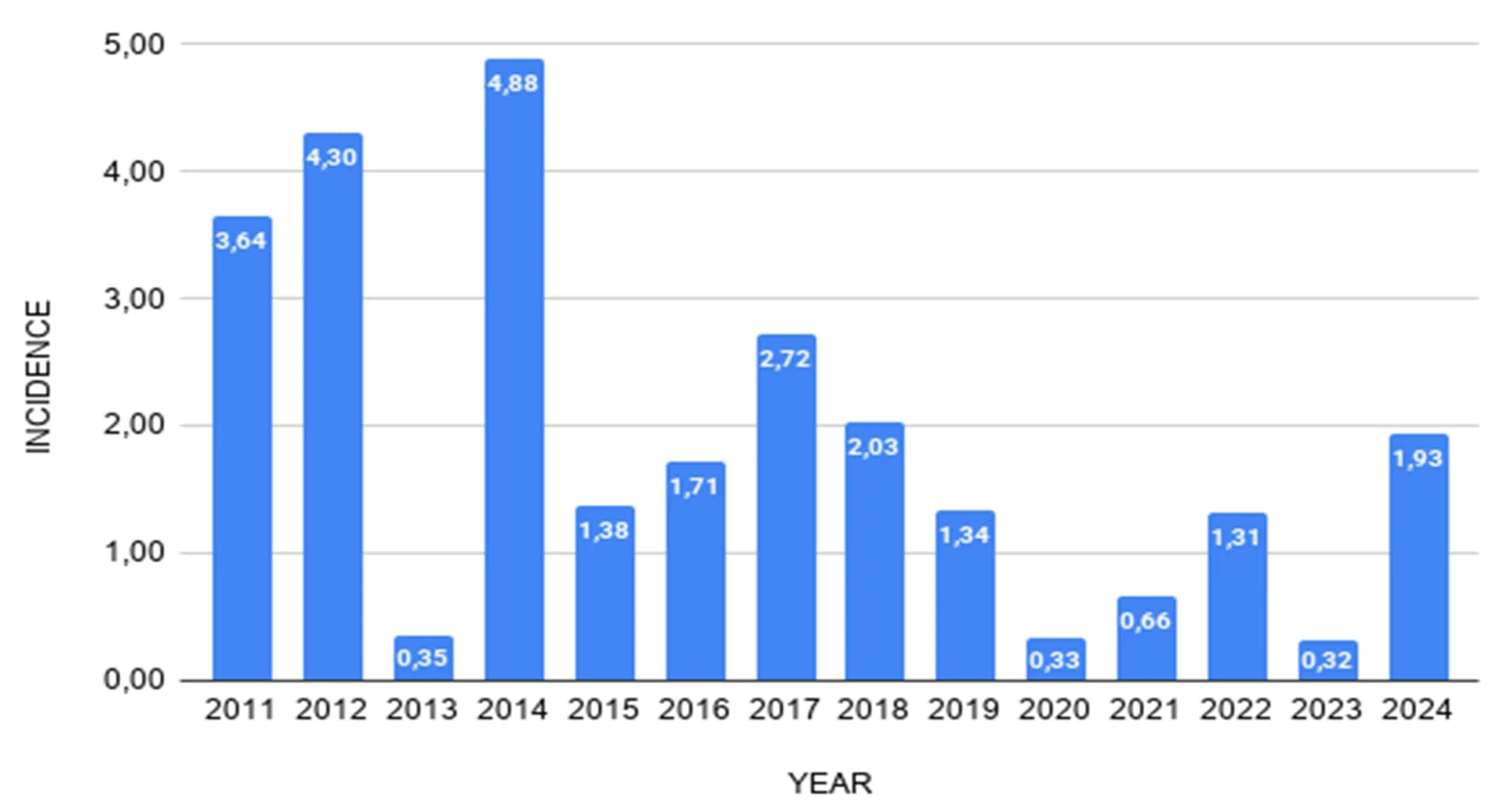
Annual incidence of leprosy in Sonora, Mexico, 2011–2024 (*n* = 78). Incidence rates are expressed as newly diagnosed cases per 1,000,000 inhabitants and were calculated using annual population estimates from CONAPO/INEGI. In 2012, the incidence rate was 4.30 cases per 1,000,000 inhabitants.

**Figure 3:**
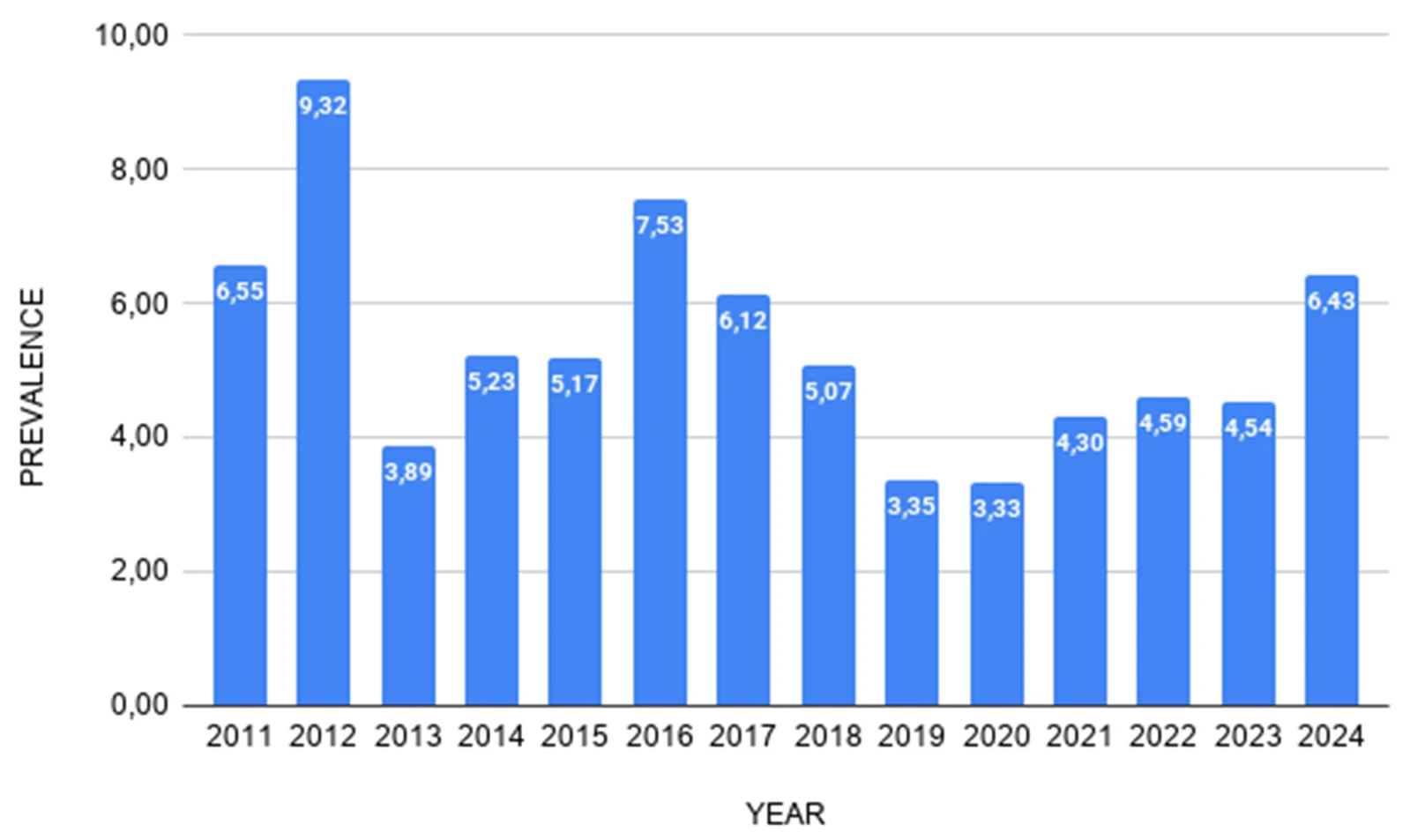
Annual prevalence of leprosy in Sonora, Mexico, 2011–2024 (*n* = 78). Prevalence rates are expressed as cases per 1,000,000 inhabitants and were calculated using annual population estimates from CONAPO/INEGI. The highest prevalence was observed in 2012, at 9.32 cases per 1,000,000 inhabitants.

**Table 1:** Sociodemographic characteristics (*n* = 75).

Variable	Category	Number (*n*)	Percentage (%)
Sex	Male	38	50.66
	Female	37	49.33
Age	0–9	0	0
	10–19	3	4
	20–29	11	14.7
	30–39	17	22.7
	40–49	19	25.3
	50–59	13	17.3
	60–69	5	6.7
	70–79	5	6.7
	80–89	2	2.7
Occupation	Employed	62	82.66
	Unemployed	13	17.33
Residence	Urban	63	84
	Rural	12	16

Data are presented as absolute frequencies (*n*) and percentages (%). Sociodemographic characteristics were available for 75 of the 78 registered cases. Residence corresponds to the urban or rural classification recorded in the state surveillance database.

**Table 2:** Clinical manifestations (*n* = 75).

Variable	Category	Number (*n*)	Percentage (%)
Hypopigmented macules	0 lesions	37	49.3
	1 lesion	0	0
	2–5 lesions	4	5.3
	>5 lesions	34	45.3
Erythematous–coppery macules	0 lesions	46	61.3
	1 lesion	3	4
	2–5 lesions	5	6.7
	>5 lesions	21	28
Infiltrated plaques	0 lesions	58	77.3
	1 lesion	0	0
	2–5 lesions	4	5.3
	>5 lesions	13	17.3
Nodules	0 lesions	45	60
	1 lesion	3	4
	2–5 lesions	5	6.7
	>5 lesions	22	29.3
Diffuse infiltration	Yes	2	2.7
	No	73	97.3
Neuritis	Yes	3	4
	No	72	96
Anesthetic areas	0 lesions	30	40
	1 lesion	1	1.3
	2–5 lesions	9	12
	>5 lesions	35	46.7
Disability at diagnosis	Grade 0	25	33.3
	Grade 1	38	50.6
	Grade 2	12	16
Leprosy reactions	No reaction	63	84
	Type 1	2	2.6
	Type 2	12	16

Data are presented as absolute frequencies (*n*) and percentages (%). Clinical manifestations include the number of cutaneous lesions, neurological involvement, disability grade at diagnosis, and leprosy reactions among the 75 patients with available clinical information.

**Table 3:** Diagnostic delay from symptom onset to diagnosis (*n* = 75).

Variable	Category	Number (*n*)	Percentage (%)
Time from symptom onset to diagnosis	0–12 months	18	24
	13–36 months	22	29.3
	37–60 months	18	24
	>60 months	17	22.7

Data are presented as absolute frequencies (*n*) and percentages (%). Diagnostic delay represents the interval between symptom onset and confirmed diagnosis among 75 patients with available data.

**Table 4:** Treatment characteristics and patient outcomes (*n* = 75).

Variable	Category	Number (*n*)	Percentage (%)
Patient outcome	Cured	43	57.3
	Under treatment/follow-up	13	17.3
	Deceased	7	9.3
	Unknown	6	8.0
	Lost to follow-up	5	6.7
	Treatment abandonment	1	1.3
Level of care for treatment	Primary	54	72.0
	Secondary	18	24.0
	Tertiary	3	4.0

Treatment characteristics and patient outcomes among leprosy patients in Sonora, Mexico, 2011–2024 (*n* = 75). Data are presented as absolute frequencies (*n*) and percentages (%). Patient outcome corresponds to the last available record, and level of care refers to the setting where treatment or follow-up was provided.

## Data Availability

The data analyzed in this study were obtained from the Secretaría de Salud and are under the custody and ownership of that institution. The authors are not authorized to make the dataset publicly available or deposit it in an open repository. Access to the data may be considered upon reasonable request and is subject to prior authorization from the Secretaría de Salud, as well as applicable institutional, ethical, and confidentiality requirements.
